# Extraventricular Choroid Plexus Papilloma: A Case Report

**DOI:** 10.7759/cureus.94360

**Published:** 2025-10-11

**Authors:** Matthew R Bennett, Brooke Martin, Coplen D Johnson, Carolina Soto-Davila, Areli K Cuevas-Ocampo, Octavio Arevalo

**Affiliations:** 1 Radiology, Louisiana State University (LSU) Health, Shreveport, USA; 2 Radiology, Louisiana State University (LSU) Health Sciences Center, Shreveport, USA; 3 Pathology, Louisiana State University (LSU) Health, Shreveport, USA; 4 Neuroradiology, Louisiana State University (LSU) Health, Shreveport, USA; 5 Neuroradiology, Louisiana State University (LSU) Health Sciences Center, Shreveport, USA

**Keywords:** atypical choroid plexus papilloma, benign brain tumor, choroid plexus papilloma, choroid plexus tumors, embryonic tumor, extra-axial lesions, hydrocephalus, neuroradiology, radiology

## Abstract

The patient is a 25-year-old female transferred from an outside facility with week-long complaints of a headache, nausea, and vomiting. Computed tomography (CT) and magnetic resonance imaging (MRI) revealed obstructive hydrocephalus and a large cystic mass of the posterior fossa. Subsequent external ventricular drain placement and surgical resection were performed. The patient was discharged six days postoperatively with resolution of symptoms. Pathological examination of the fleshy component within the cystic structure after surgical resection was relatively inconclusive, with findings suggestive of a choroid plexus tumor. However, the lesion demonstrates the immunohistochemical characteristics of a choroid plexus papilloma (CPP) but with a cystic structure surrounding it.

CPPs are rare and typically benign tumors that originate from the choroid plexus, which is responsible for cerebrospinal fluid (CSF) production. These growths can occur throughout the ventricular system of the brain, more commonly in the lateral ventricles and less commonly in the third and fourth ventricles. Differential diagnosis based on imaging is broad due to the shared visual characteristics of various brain tumors. Consequently, immunohistochemical markers are essential for the identification and diagnosis of CPPs. In the case of our patient, the small enhancing nodule is unusual in that it is surrounded by a large cystic lesion.

We present the unusual case of a cystic tumor of the posterior fossa with a small nodular component located within, suggestive of a CPP by immunohistochemical and morphological characteristics. A multidisciplinary diagnostic approach is necessary to exact a proper classification of lesions such as this one, including identification by neuroradiology, resection by neurosurgery, and immunohistochemical and morphological analysis by pathology.

## Introduction

Choroid plexus papillomas (CPP) are neuroectodermal tumors that originate from dysregulation of choroid epithelial cells within the ventricles of the brain; however, in rare cases, these tumors can develop in extraventricular locations, creating a complicating radiographic and clinical diagnosis. These tumors comprise less than 1% of all brain tumors and are mostly observed in pediatric populations (70% in patients less than two years old) [1]. From a population-based study in the US, the age-adjusted incidence rate of CPPs was 0.034 per 100,000 people [2].

Clinical findings are often consistent with those of mass effect and increased intracranial pressure, such as nausea, vomiting, and headache. Typical locations for these tumors vary based on the patient's age: supratentorial within the lateral ventricles in infants and infratentorial in the fourth ventricle in young adults [3,4]. We present the case of a cystic presentation of a CPP in the posterior fossa of a young adult.

Embryologically, CPPs originate from the choroid plexus after its formation from the neuroectoderm. The neural tube itself contains a ventricular zone, which is lined with ependymal cells. The lining of these cells forms the roof plate of the neural tube, which then comes in contact with invaginating mesenchyme, allowing blood flow and differentiation into specialized ependymal cells to form the choroid plexus epithelium. The presentation of a CPP is typically located in the fourth ventricle in adults, since the choroid plexus of the lateral ventricles is proportionally smaller [3,4]. Differential diagnosis is broad due to common characteristics of other brain tumors, including medulloblastomas, atypical teratoid/rhabdoid tumors, intraventricular meningiomas, ependymomas, central neurocytomas, and exophytic gliomas [5]. Although imaging may not necessarily narrow the differential, immunohistochemical markers can be used to further characterize the tumor.

CPPs are characterized histologically by papillary fronds lined by bland columnar epithelium, which typically stain positive for cytokeratin, vimentin, podoplanin, and S-100 protein [6]. Clinicopathologic associations have been made with age, suggesting that young adults typically express more glial fibrillary acidic protein (GFAP) and transthyretin (TTR) than younger patients. Although uncommon, CPPs may possess malignant characteristics like high mitotic activity, nuclear pleomorphism, high cellularity, papillary growth pattern blurring, or necrosis [7].

The gold standard of treatment is surgical resection in these patients if the anatomical position permits. Delayed intervention may lead to worsening hydrocephalus, leading to mass effect on cerebral structures. Severe presentations involve seizures, cognitive deficit, or even subarachnoid hemorrhage. Since these tumors are often highly vascular, there is a high intraoperative risk of bleeding, leading to a pediatric perioperative mortality of 12% [8].

## Case presentation

**Figure 1:**
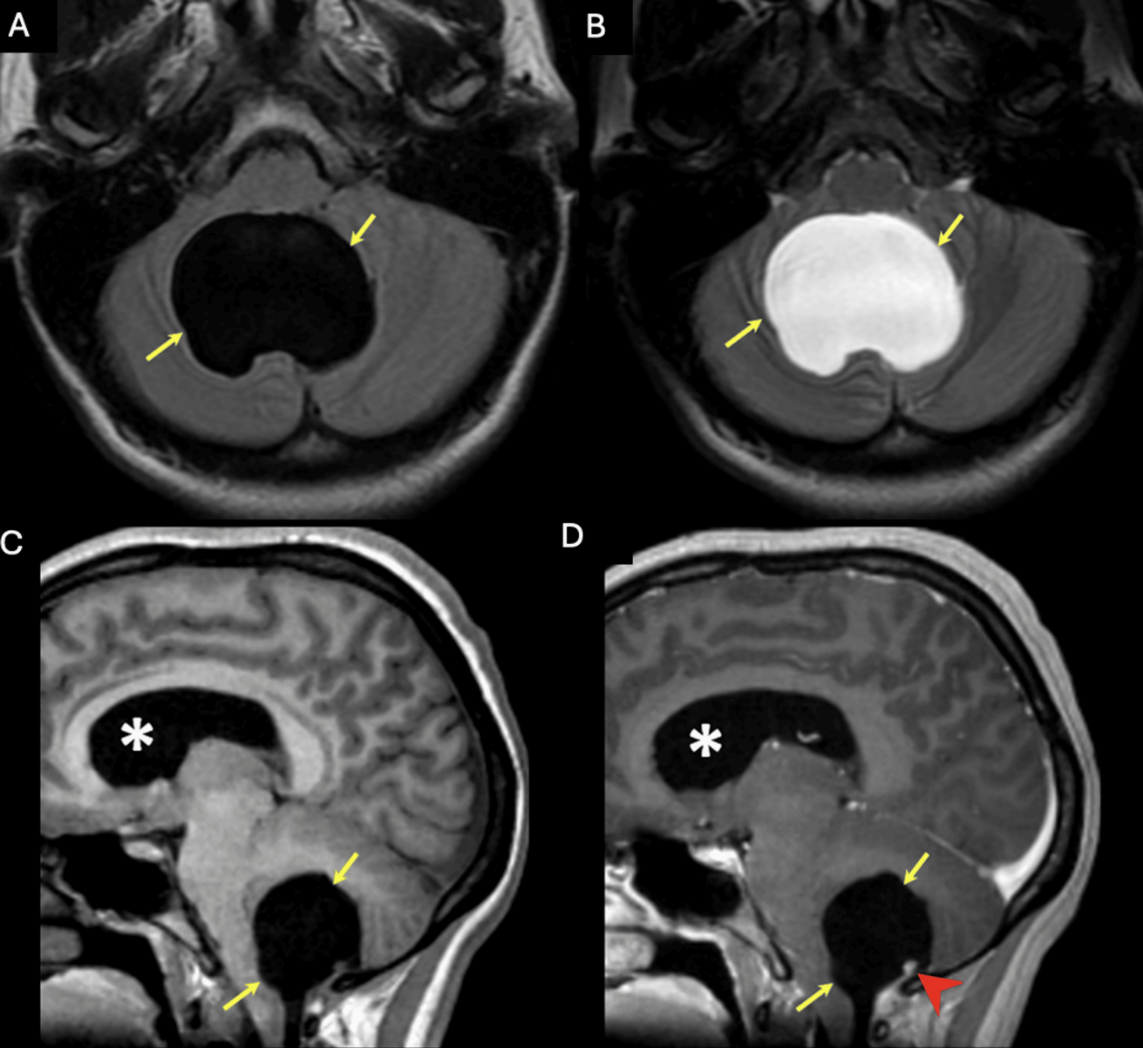
A) Axial FLAIR and B) axial T2WI MRI of the brain showed a cystic extra-axial lesion located at the inferior aspect of the cisterna magna (yellow arrows); C) Sagittal T1WI without contrast and D) sagittal T1WI postcontrast showed an enhancing nodule (red arrowhead) and dilation of the lateral ventricle (asterisk) secondary to obstructive hydrocephalus

**Figure 2:**
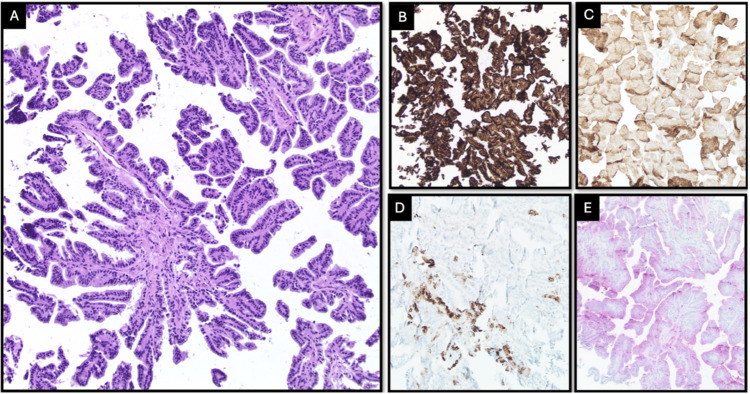
A) Cystic papillary lesion composed of cuboidal cells arranged in papillary fronds with well-formed fibrovascular cores without nuclear atypia at intermediate magnification or 100x H&E; B) The cells are positive for cytokeratin CAM5.2 and show C) focal positivity for CK7, D) positivity for S100 and glial fibrillary acidic protein (GFAP) (not shown), and E) membranous staining for synaptophysin, consistent with a choroid plexus tumor phenotype

**Figure 3:**
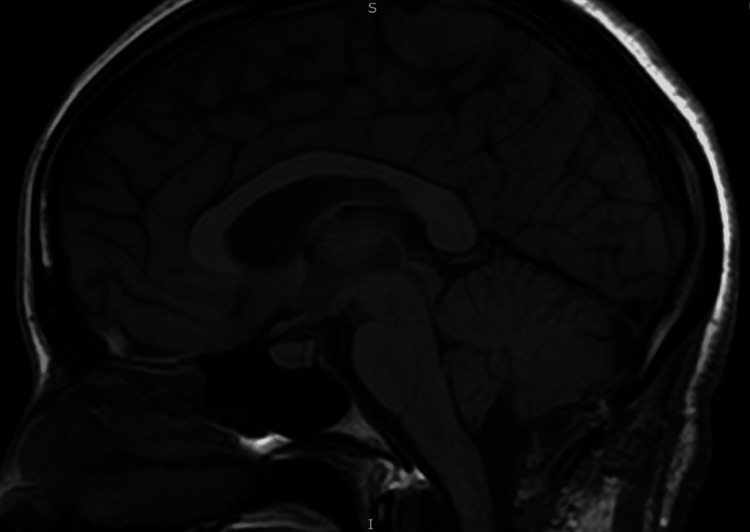
Post-surgical changes of posterior fossa cystic mass resection are demonstrated with normal lateral, third, and fourth ventricular spaces. Hydrocephalus was resolved by the surgical intervention. In addition, no hyperechoic tumors were observed in the cerebellum. However, residual damage was observed in the ventral cerebellum, and a continued enlargement of the cisterna magna was present.

A 25-year-old female presented to the emergency room with complaints of generalized headaches, nausea, and vomiting that had occurred for the last week. She indicated that she has had these symptoms previously and that the last episode was four months ago. Her symptoms usually regress with over-the-counter pain relievers and head massaging. The neurological examination was nominal for cranial nerve symptoms and motor or sensory deficits. The visual field was normal. The pupils were round, responsive, and equal and reactive to light. No seizure activity was noted. Cerebellar signs were noted on the physical exam, with a positive Romberg, a broad-based and unsteady gait with truncal ataxia, and right-sided dysmetria on finger-to-nose and heel-to-shin tests. MRI revealed a large cystic mass in the posterior fossa, causing a significant amount of mass effect on the brainstem in addition to obstructive hydrocephalus (Figure 1). Neurosurgery was consulted for extraventricular drain placement for drainage of the cystic space and subsequent mass resection. A suboccipital craniotomy was performed to access the posterior fossa. Following the dural opening, the cystic space became immediately apparent. Microscopic examination showed that the walls of the cyst were distinct from the overlying arachnoid and underlying brainstem and cerebellum. On careful inspection of the cyst wall, a tiny flesh-colored nodular component became apparent. Both the cyst wall and portion of the nodule were sent for frozen pathology. These findings revealed characteristics of a CPP (Figure 2). The patient had an unremarkable six-day postoperative course and was discharged with full resolution of her cerebellar signs and hydrocephalus-related symptoms. An MRI performed a week later revealed normal post-surgical changes with no recurrence of the cystic extraventricular mass, although there is still mild cerebellar damage and an increase in the size of the cisterna magna (Figure 3).

## Discussion

CPPs are rare and typically benign tumors originating from the choroid plexus, which is responsible for the production of CSF. These tumors can occur throughout the ventricular system of the brain, more commonly in the lateral ventricles and less commonly in the third and fourth ventricles [9]. Many differential diagnoses can account for the radiological imaging in this case. The use of immunohistochemical analysis can be beneficial in providing an accurate diagnosis. Consequently, immunohistochemical markers are essential in the identification and diagnosis of CPPs, such as cytokeratin, vimentin, podoplanin, and S-100 protein [6]. In the case of our patient, the small enhancing nodule is unusual in that it is surrounded by a large cystic growth with a similar phenotype. The World Health Organization currently classifies such growths as papillomas (grade I), atypical tumors (grade II), or carcinomas (grade III). As mentioned above, the grading of these tumors is based primarily upon the mitotic activity and the presence of four or more malignant histopathological characteristics. Of note, some studies have suggested that the epigenetic profiling of these tumors into three clusters indicates prognosis for these patients: cluster one involves supratentorial pediatric choroid plexus tumors like benign papillomas and atypical tumors, cluster two involves infratentorial adult CPPs and atypical tumors, and cluster three involves supratentorial pediatric choroid plexus tumors, including papilloma, atypical tumors, and carcinomas [10].

A large cystic mass, histology consistent with an endodermal cyst, surrounding a small enhancing nodule, consistent with CPP, in our case, is unusual. Five cases of peritumoral cystic growth of the posterior fossa are present in the literature, with those present having a much smaller cystic growth than the one in this case [11-15]. Craniospinal cystic dissemination of CPPs has also been noted. Additional mixed tissue CPPs currently present in the literature include mature cartilage, consistent with a chondroma, and those with features mimicking a hemangioblastoma [15].

Typical CPPs will show positivity for cytokeratin, TTR, S100, and variably GFAP and synaptophysin positivity [16-18]. Histologically, our patient’s tumor demonstrated the classic morphology of CPP but with expanded immunophenotypic features, most notably membranous synaptophysin positivity. Synaptophysin immunoreactivity is described in normal choroid plexus epithelium and in choroid plexus papillomas and carcinomas, though expression is variably present (not always strong or diffuse) [6]. Our patient shows some consistency with the generalized markers leading to the diagnosis of CPP, but the aberrancy arises with the cystic component. These factors alone are not diagnostic of malignancy, but they highlight that CPPs can show variable immunohistochemistry beyond the classic profile. The lack of atypia or mitoses keeps it in the benign CPP category.

This case report contains certain limitations, notably the uncertainty regarding the presentation and etiology of the tumor. It is unclear if the tumor in this case is congenital since the symptoms did not occur until the patient was 25 years old. It is possible that the pathology could have been present throughout her life, but she did not exhibit symptoms until later in life. The lack of genetic testing in this case poses another etiologic challenge. Another possibility is that the tumor spontaneously developed in the patient when the patient was an adult. The onset of symptoms, in this case, is likely to have occurred once the tumor blocked CSF flow, causing an increase in intracranial pressure and hydrocephalus. Another consideration is limited access to healthcare, which could have contributed to the magnitude of presentation and complicated management. Due to this presentation, it is relatively unclear as to whether the tumor is congenital despite the immunohistochemical findings. Follow-up monitoring should be recommended for a recurrence of the tumor, a recurrence of hydrocephalus, or infections that were introduced during the surgical procedure, as these sequelae are common [19,20].

## Conclusions

In summary, CPPs are rare, typically benign neuroectodermal tumors that pose a diagnostic challenge due to their overlapping radiologic and immunohistochemical features with other intracranial tumors. A careful combination of imaging, histopathologic confirmation, tumor grading, and maximal safe resection underpins optimal management of CPPs, with prognosis largely dependent on completeness of excision and tumor grade. The present case is notable for its large cystic component consistent with an endodermal cyst enveloping a smaller nodule of CPP, an unusual mixed tumor given the distinct embryological origins of the two entities. This coexistence raises the possibility of a shared developmental pathway or pluripotent progenitor cell, a hypothesis that requires further molecular investigation. While this finding is atypical, the absence of nuclear atypia, increased mitotic activity, or other malignant histopathological features supports classification as a WHO grade I papilloma. Future studies can be aimed at identifying this molecular origin as well as pathological markers that are expressed among the CPP variants. Our findings demonstrate that integration of morphology with imaging can enhance the accuracy of diagnosis for CPP.

## References

[1] Boyd MC, Steinbok P (1987). Choroid plexus tumors: problems in diagnosis and management. J Neurosurg.

[2] Takaoka K, Cioffi G, Waite KA (2023). Incidence and survival of choroid plexus tumors in the United States. Neurooncol Pract.

[3] Dash C, Moorthy S, Garg K (2019). Management of choroid plexus tumors in infants and young children up to 4 years of age: an institutional experience. World Neurosurg.

[4] Prasad GL, Mahapatra AK (2015). Case series of choroid plexus papilloma in children at uncommon locations and review of the literature. Surg Neurol Int.

[5] (2024). Choroid plexus papilloma. https://radiopaedia.org/articles/choroid-plexus-papilloma-1.

[6] Ikota H, Tanaka Y, Yokoo H, Nakazato Y (2011). Clinicopathological and immunohistochemical study of 20 choroid plexus tumors: their histological diversity and the expression of markers useful for differentiation from metastatic cancer. Brain Tumor Pathol.

[7] Jeibmann A, Hasselblatt M, Gerss J (2006). Prognostic implications of atypical histologic features in choroid plexus papilloma. J Neuropathol Exp Neurol.

[8] Toescu SM, James G, Phipps K, Jeelani O, Thompson D, Hayward R, Aquilina K (2019). Intracranial neoplasms in the first year of life: results of a third cohort of patients from a single institution. Neurosurgery.

[9] Anderson MD, Theeler BJ, Penas-Prado M, Groves MD, Yung WK (2013). Bevacizumab use in disseminated choroid plexus papilloma. J Neurooncol.

[10] Thomas C, Metrock K, Kordes U, Hasselblatt M, Dhall G (2020). Epigenetics impacts upon prognosis and clinical management of choroid plexus tumors. J Neurooncol.

[11] van Swieten JC, Thomeer RT, Vielvoye GJ, Bots GT (1987). Choroid plexus papilloma in the posterior fossa. Surg Neurol.

[12] Girardot C, Boukobza M, Lamoureux JP (1990). Choroid plexus papillomas of the posterior fossa in adults: MR imaging and gadolinium enhancement. Report of four cases and review of the literature. J Neuroradiol.

[13] Luo W, Liu H, Li J, Yang J, Xu Y (2016). Choroid plexus papillomas of the cerebellopontine angle. World Neurosurg.

[14] Matsushita S, Shimono T, Goto T, Doishita S, Kuwae Y, Miki Y (2019). Posterior fossa choroid plexus papilloma with focal ependymal differentiation in an adult patient: a case report and literature review. Radiol Case Rep.

[15] Johnson GW, Mian AY, Dahiya S, Rich KM, Chicoine MR, Limbrick DD (2022). Cystic dissemination of choroid plexus papilloma: illustrative cases. J Neurosurg Case Lessons.

[16] García-Valtuille R, Abascal F, García-Valtuille AI (2000). Adult choroid plexus papilloma of the posterior fossa mimicking a hemangioblastoma. Case report. J Neurosurg.

[17] Wolff JE, Sajedi M, Brant R, Coppes MJ, Egeler RM (2002). Choroid plexus tumours. Br J Cancer.

[18] Khade S, Shenoy A (2018). Ectopic choroid plexus papilloma. Asian J Neurosurg.

[19] Hosmann A, Hinker F, Dorfer C (2019). Management of choroid plexus tumors-an institutional experience. Acta Neurochir (Wien).

[20] Kernan WN, Adib SD, Vogel TW (2023). Perioperative complications in intracranial tumor surgery: current perspectives. Surg Neurol Int.

